# Supplementary material for: Mutations in L-type amino acid transporter-2 support SLC7A8 as a novel gene involved in age-related hearing loss
Source: eLife. 2018 Jan 22;7:e31511. doi: 10.7554/eLife.31511 (PMC5811215; doi:10.7554/eLife.31511)
Supplement: Transparent reporting form [file elife-31511-transrepform.docx]

***eLife’s* transparent reporting form**

We encourage authors to provide detailed information *within their submission* to facilitate the interpretation and replication of experiments. Authors can upload supporting documentation to indicate the use of appropriate reporting guidelines for health-related research (see [EQUATOR Network](http://www.equator-network.org/%20)), life science research (see the [BioSharing Information Resource](https://biosharing.org/)), or the [ARRIVE guidelines](http://www.plosbiology.org/article/info:doi/10.1371/journal.pbio.1000412) for reporting work involving animal research. Where applicable, authors should refer to any relevant reporting standards documents in this form.

If you have any questions, please consult our Journal Policies and/or contact us: [editorial@elifesciences.org](mailto:editorial@elifesciences.org).

**Sample-size estimation**

- You should state whether an appropriate sample size was computed when the study was being designed
- You should state the statistical method of sample size computation and any required assumptions
- If no explicit power analysis was used, you should describe how you decided what sample (replicate) size (number) to use

Please outline where this information can be found within the submission (e.g., sections or figure legends), or explain why this information doesn’t apply to your submission:

All figures legends include the number, age and background of the mice used to plot the data. Moreover, a summary table is included below to facilitate the quick check.

**Replicates**

- You should report how often each experiment was performed
- You should include a definition of biological versus technical replication
- The data obtained should be provided and sufficient information should be provided to indicate the number of independent biological and/or technical replicates
- If you encountered any outliers, you should describe how these were handled
- Criteria for exclusion/inclusion of data should be clearly stated
- High-throughput sequence data should be uploaded before submission, with a private link for reviewers provided (these are available from both GEO and ArrayExpress)

Please outline where this information can be found within the submission (e.g., sections or figure legends), or explain why this information doesn’t apply to your submission:

Only experiments that had been technical replicated by at least 3 different biological samples were included. Moreover, a summary table is included below to facilitate the quick check.

**Statistical reporting**

- Statistical analysis methods should be described and justified
- Raw data should be presented in figures whenever informative to do so (typically when N per group is less than 10)
- For each experiment, you should identify the statistical tests used, exact values of N, definitions of center, methods of multiple test correction, and dispersion and precision measures (e.g., mean, median, SD, SEM, confidence intervals; and, for the major substantive results, a measure of effect size (e.g., Pearson's r, Cohen's d)
- Report exact p-values wherever possible alongside the summary statistics and 95% confidence intervals. These should be reported for all key questions and not only when the p-value is less than 0.05.

Please outline where this information can be found within the submission (e.g., sections or figure legends), or explain why this information doesn’t apply to your submission:

All figures legends and methods include the statistical test used. Moreover, a summary table is included below to facilitate the quick check.

(For large datasets, or papers with a very large number of statistical tests, you may upload a single table file with tests, Ns, etc., with reference to sections in the manuscript.)

**Group allocation**

- Indicate how samples were allocated into experimental groups (in the case of clinical studies, please specify allocation to treatment method); if randomization was used, please also state if restricted randomization was applied
- Indicate if masking was used during group allocation, data collection and/or data analysis

Please outline where this information can be found within the submission (e.g., sections or figure legends), or explain why this information doesn’t apply to your submission:

Mice housing allocation was random. Experiments procedures and samples acquisition timing were performed using the individual animal code, genotype blinded.

**Additional data files (“source data”)**

- We encourage you to upload relevant additional data files, such as numerical data that are represented as a graph in a figure, or as a summary table
- Where provided, these should be in the most useful format, and they can be uploaded as “Source data” files linked to a main figure or table
- Include model definition files including the full list of parameters used
- Include code used for data analysis (e.g., R, MatLab)
- Avoid stating that data files are “available upon request”

Please indicate the figures or tables for which source data files have been provided:

Real Time PCR raw data from Fig 2D and E, and Supp. Figure S7 are provided.

The WGS data are available through the EGA website (<https://www.ebi.ac.uk/ega/home>).

Table summarizing biological replicates, gender and genetic background of mice.

| **Figure** | **#** | **Biological Samples** | **Sex** | **Age months** | **Background** | **Technical Replicates** | **Statistical reporting** |
| --- | --- | --- | --- | --- | --- | --- | --- |
| Figure 1 A | 6 | wild type | male | 2 to 3 | C57BL6/J-129Sv | 2 | N/A |
|  | 6 | Lat2-/- |  |  |  | 2 |  |
|  | 6 | wild type | female |  |  | 2 |  |
|  | 6 | Lat2-/- |  |  |  | 2 |  |
| Figure 1 B | 19 | wild type | male | 4 to 7 | C57BL6/J-129Sv | 2 | Paired Student’s t-test |
|  | 15 | Lat2-/- |  |  |  | 2 |  |
| Figure 1 C to F and Figure 1- supplement 4 | 5 | wild type | male | 4 to 6 | C57BL6/J-129Sv | 1 | ANOVA test |
|  | 5 | hetero |  |  |  | 1 |  |
|  | 8 | Lat2-/- |  |  |  | 1 |  |
|  | 0 | wild type | female |  |  | 1 |  |
|  | 0 | hetero |  |  |  | 1 |  |
|  | 0 | Lat2-/- |  |  |  | 1 |  |
|  | 3 | wild type | male | 7 to 13 |  | 1 |  |
|  | 4 | hetero |  |  |  | 1 |  |
|  | 4 | Lat2-/- |  |  |  | 1 |  |
|  | 3 | wild type | female |  |  | 1 |  |
|  | 3 | hetero |  |  |  | 1 |  |
|  | 3 | Lat2-/- |  |  |  | 1 |  |
| Figure 2 A to C | 2 | wild type | male | 4 to 7 | C57BL6/J-129Sv | 4 | Unpaired Student’s t-test |
|  | 3 | hetero |  |  |  | 4 |  |
|  | 2 | Lat2-/- |  |  |  | 4 |  |
|  | 2 | wild type | male |  | C57BL6/J | 4 |  |
|  | 0 | hetero |  |  |  | 4 |  |
|  | 2 | Lat2-/- |  |  |  | 4 |  |
| Figure 2 D | 3 | wild type | male | 2 | CBA | 4 | Unpaired Student’s t-test |
|  | 3 |  |  | 12 |  | 4 |  |
| Figure 2-supplement 1A | 3 | wild type | male | E15.5 | MF1/129Sv | 3 | Unpaired Student’s t-test |
|  | 3 |  |  | E18.5 |  | 3 |  |
|  | 3 |  |  | 5 days |  | 3 |  |
|  | 3 |  |  | 15 days |  | 3 |  |
|  | 3 |  |  | 1 to 2 |  | 3 |  |
|  | 3 |  |  | 5 days |  | 3 |  |
|  | 3 |  |  | 12 |  | 3 |  |
| Figure 3 A to C and Figure 3-supplement 1 | 2 | wild type | male | 4 to 7 | C57BL6/J-129Sv | 4 | Unpaired Student’s t-test |
|  | 3 | hetero |  |  |  | 4 |  |
|  | 2 | Lat2-/- |  |  |  | 4 |  |
|  | 2 | wild type | male |  | C57BL6/J | 4 |  |
|  | 0 | hetero |  |  |  | 4 |  |
|  | 2 | Lat2-/- |  |  |  | 4 |  |
| Figure 3D and Figure 2-Supplement 1B and C | 3 | wild type | male | 3 | C57BL6/J | 3 | Unpaired Student’s t-test |
|  | 3 | hetero |  |  |  | 3 |  |
|  | 3 | Lat2-/- |  |  |  | 3 |  |
|  | 3 | wild type |  | 7 |  | 3 |  |
|  | 3 | hetero |  |  |  | 3 |  |
|  | 3 | Lat2-/- |  |  |  | 3 |  |
| Figure 4 and Figure 4-supplement 1 | 2 | wild type | male | 4 to 7 | C57BL6/J-129Sv | 4 | Unpaired Student’s t-test |
|  | 3 | hetero |  |  |  | 4 |  |
|  | 2 | Lat2-/- |  |  |  | 4 |  |
|  | 2 | wild type | male |  | C57BL6/J | 4 |  |
|  | 0 | hetero |  |  |  | 4 |  |
|  | 2 | Lat2-/- |  |  |  | 4 |  |
| Figure 5 | 3 | wt | HeLa Cells | |  | 4 | Unpaired Student’s t-test |
|  | 3 | V302I |  |  |  | 4 |  |
|  | 3 | T402M |  |  |  | 4 |  |
|  | 3 | R418C |  |  |  | 4 |  |
|  | 3 | V460E |  |  |  | 4 |  |
|  | 3 | R185L |  |  |  | 4 |  |
|  | 3 | R8P |  |  |  | 4 |  |
|  | 3 | A94T |  |  |  | 4 |  |
| Figure 1-supplement 2 | 4 | wild type | male | 4 to 7 | C57BL6/J-129Sv | 2 | N/A |
|  | 4 | Lat2-/- |  |  |  | 2 |  |
| Figure 2- supplement 3A and G | 16 | wild type | male | 4 to 7 | C57BL6/J-129Sv | 2 | Paired Student’s t-test |
|  | 14 | Lat2-/- |  |  |  | 2 |  |
| Figure 2- supplement 3B and C | 8 | wild type | male | 4 to 7 | C57BL6/J-129Sv | 2 | Paired Student’s t-test |
|  | 8 | Lat2-/- |  |  |  | 2 |  |
| Figure 2- supplement 3D and F | 5 | wild type | male | 4 to 7 | C57BL6/J-129Sv | 2 | Paired Student’s t-test |
|  | 8 | Lat2-/- |  |  |  | 2 |  |
| Figure 2- supplement 3H | 3 | wild type | male | 2 to 3 | C57BL6/J-129Sv | 2 | N/A |
|  | 3 | Lat2-/- |  |  |  | 2 |  |
|  | 3 | wild type | female |  |  | 2 |  |
|  | 3 | Lat2-/- |  |  |  | 2 |  |
| Figure 2- supplement 3I | 4 | wild type | male | 2 to 3 | C57BL6/J-129Sv | 1 | N/A |
|  | 5 | Lat2-/- |  |  |  | 1 |  |
| Figure 1- supplement 4 | 11 | wild type | male | 4 to 6 | C57BL6/J | 1 | ANOVA test |
|  | 2 | hetero |  |  |  | 1 |  |
|  | 7 | Lat2-/- |  |  |  | 1 |  |
|  | 9 | wild type | female |  |  | 1 |  |
|  | 3 | hetero |  |  |  | 1 |  |
|  | 5 | Lat2-/- |  |  |  | 1 |  |
|  | 4 | wild type | male | 7 to 13 |  | 1 |  |
|  | 7 | Lat2-/- |  |  |  | 1 |  |
|  | 5 | wild type | female |  |  | 1 |  |
|  | 6 | Lat2-/- |  |  |  | 1 |  |
| Figure 2- supplement 2 | 19 | wild type | male | <2.5, 3.5 and 5 | C57BL6/J | 1 | ANOVA test |
|  | 13 | hetero |  |  |  | 1 |  |
|  | 23 | Lat2-/- |  |  |  | 1 |  |
